# Supplementary figures and images for: Intrinsically disordered proteins and structured proteins with intrinsically disordered regions have different functional roles in the cell
Source: PLoS One. 2019 Aug 19;14(8):e0217889. doi: 10.1371/journal.pone.0217889 (PMC6699704; doi:10.1371/journal.pone.0217889)

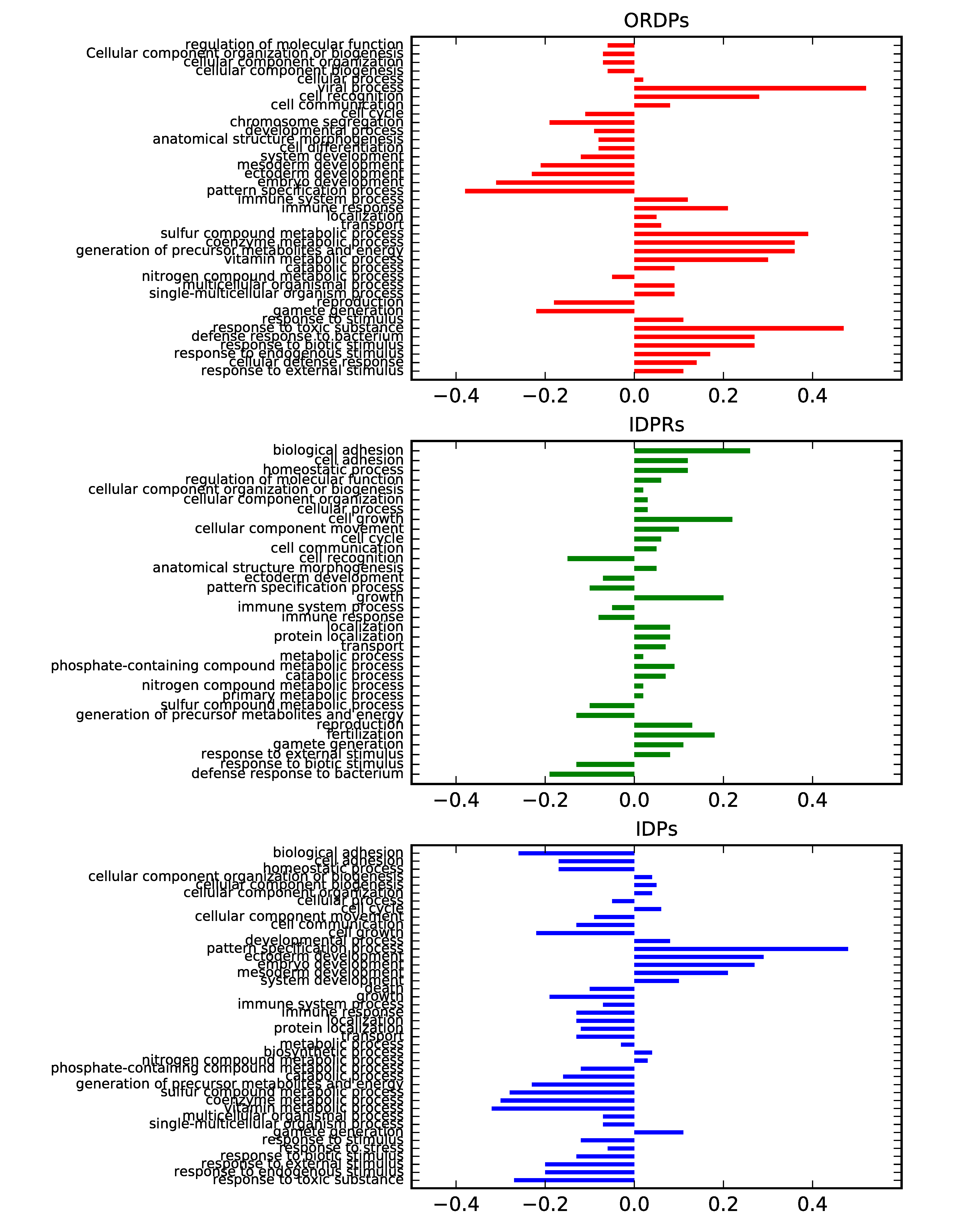

Supplement: S1 Fig — Bar charts of the normalized differential occurrence of ORDPs, IDPRs, and IDPs in various biological processes, with respect to the human proteome (the reference). Only statistically significant differences are reported (p-value <0.05). (TIF) [file pone.0217889.s001.tif]

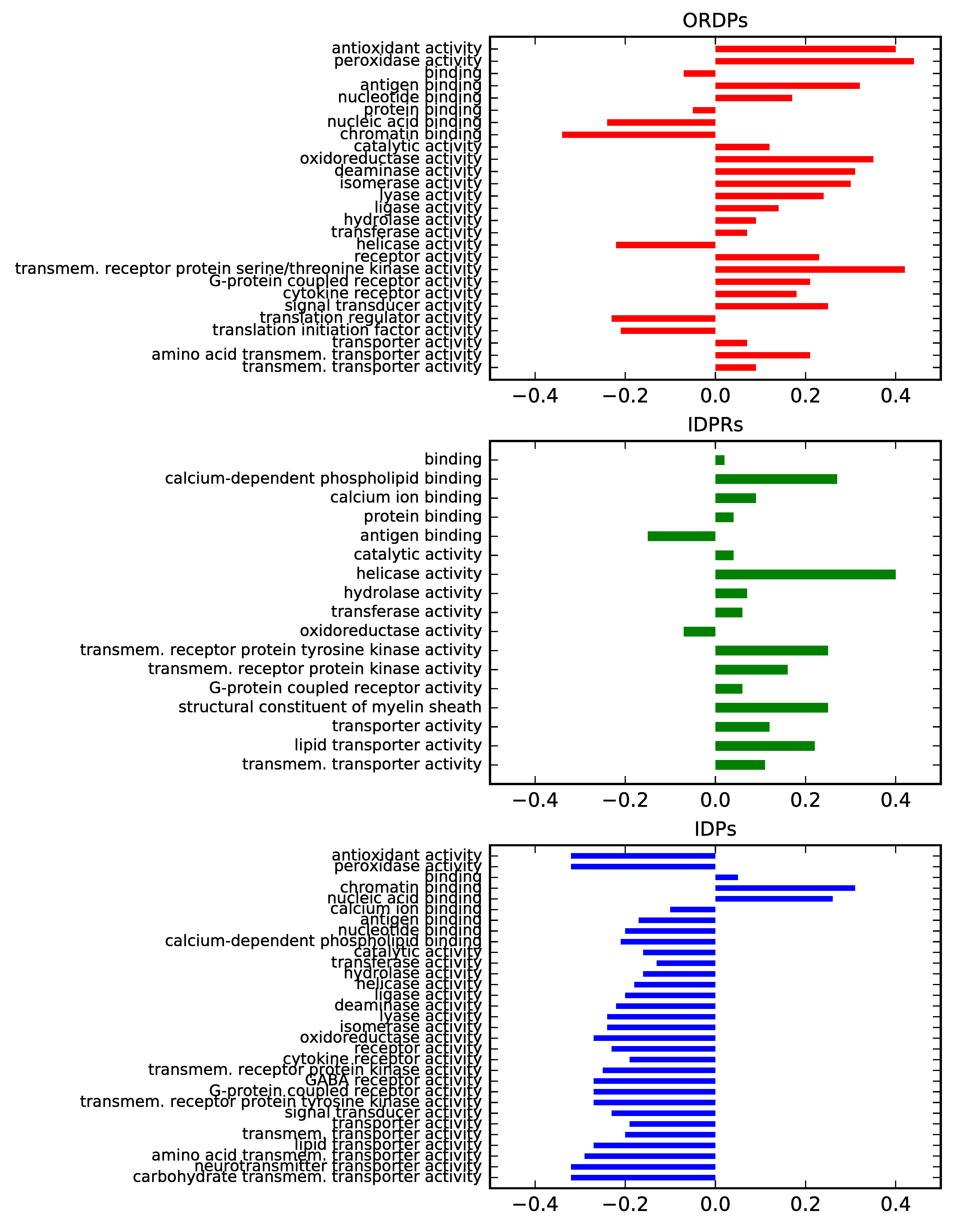

Supplement: S2 Fig — Bar charts of the normalized differential occurrence of ORDPs, IDPRs, and IDPs in various molecular functions, with respect to the human proteome (the reference). Only statistically significant differences are reported (p-value <0.05). (TIF) [file pone.0217889.s002.tif]

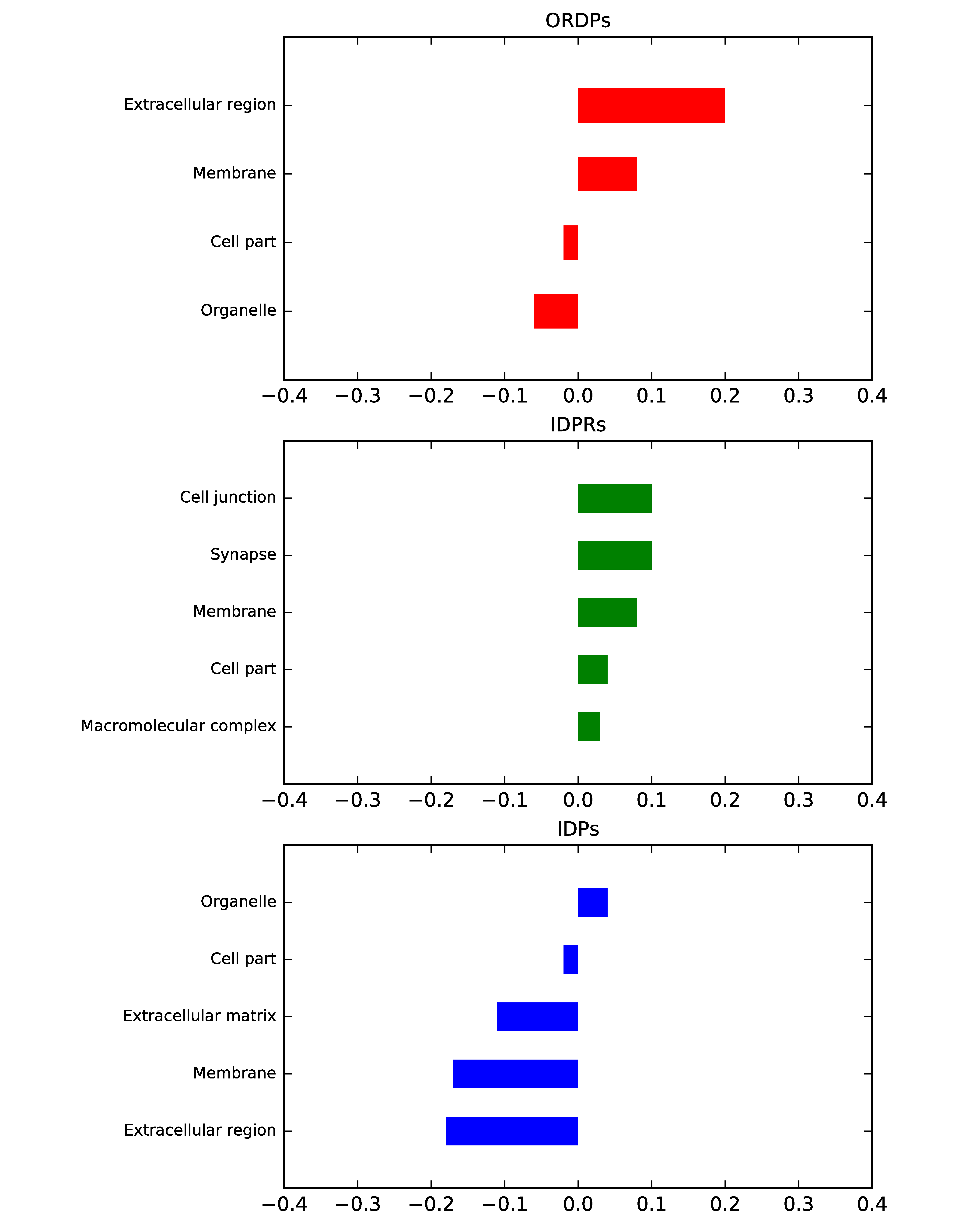

Supplement: S3 Fig — Bar charts of the normalized differential occurrence of ORDPs, IDPRs, and IDPs in various cellular components, with respect to the human proteome (the reference). Only statistically significant differences are reported (p-value <0.05). (TIF) [file pone.0217889.s003.tif]
