# Supplementary material for: Intrinsically disordered proteins and structured proteins with intrinsically disordered regions have different functional roles in the cell
Source: PLoS One. 2019 Aug 19;14(8):e0217889. doi: 10.1371/journal.pone.0217889 (PMC6699704; doi:10.1371/journal.pone.0217889)
Supplement: S1 Table — (DOCX) [file pone.0217889.s004.docx]

| Biological process | ORDPs | IDPRs | IDPs |
| --- | --- | --- | --- |
| ORDPs | 0.00 | 0.04 | 0.07 |
| IDPRs |  | 0.00 | 0.06 |
| IDPs |  |  | 0.00 |
| Molecular function | ORDPs | IDPRs | IDPs |
| ORDPs | 0.00 | 0.10 | 0.37 |
| IDPRs |  | 0.00 | 0.28 |
| IDPs |  |  | 0.00 |
| Cellular component | ORDPs | IDPRs | IDPs |
| ORPs | 0.00 | 0.03 | 0.14 |
| IDPRs |  | 0.00 | 0.13 |
| IDPs |  |  | 0.00 |
| Protein classes | ORDPs | IDPRs | IDPs |
| ORPs | 0.00 | 0.08 | 0.29 |
| IDPRs |  | 0.00 | 0.24 |
| IDPs |  |  | 0.00 |

**Table S1. Distance matrices between ORDPs, IDPRs and IDPs for different functional roles.**
