# Supplementary material for: Intrinsically disordered proteins and structured proteins with intrinsically disordered regions have different functional roles in the cell
Source: PLoS One. 2019 Aug 19;14(8):e0217889. doi: 10.1371/journal.pone.0217889 (PMC6699704; doi:10.1371/journal.pone.0217889)
Supplement: S4 Table — The asterix near some molecular function indicates that the over-representation test is not statistically significant. (DOCX) [file pone.0217889.s007.docx]

**Table S4. Over-representation and under-representation of the protein variants in the molecular functions.** The asterix near some molecular function indicates that the over-representation test is not statistically significant.
