# Supplementary material for: Intrinsically disordered proteins and structured proteins with intrinsically disordered regions have different functional roles in the cell
Source: PLoS One. 2019 Aug 19;14(8):e0217889. doi: 10.1371/journal.pone.0217889 (PMC6699704; doi:10.1371/journal.pone.0217889)
Supplement: S7 Table — The biological processes for which the enrichment test is not statistically significant are reported in red. (DOCX) [file pone.0217889.s010.docx]

**Table S7. Enrichment of the protein variants in the biological processes.** The biological processes for which the enrichment test is not statistically significant are reported in red.
