# Supplementary material for: Intrinsically disordered proteins and structured proteins with intrinsically disordered regions have different functional roles in the cell
Source: PLoS One. 2019 Aug 19;14(8):e0217889. doi: 10.1371/journal.pone.0217889 (PMC6699704; doi:10.1371/journal.pone.0217889)
Supplement: S1 Text — (DOCX) [file pone.0217889.s015.docx]

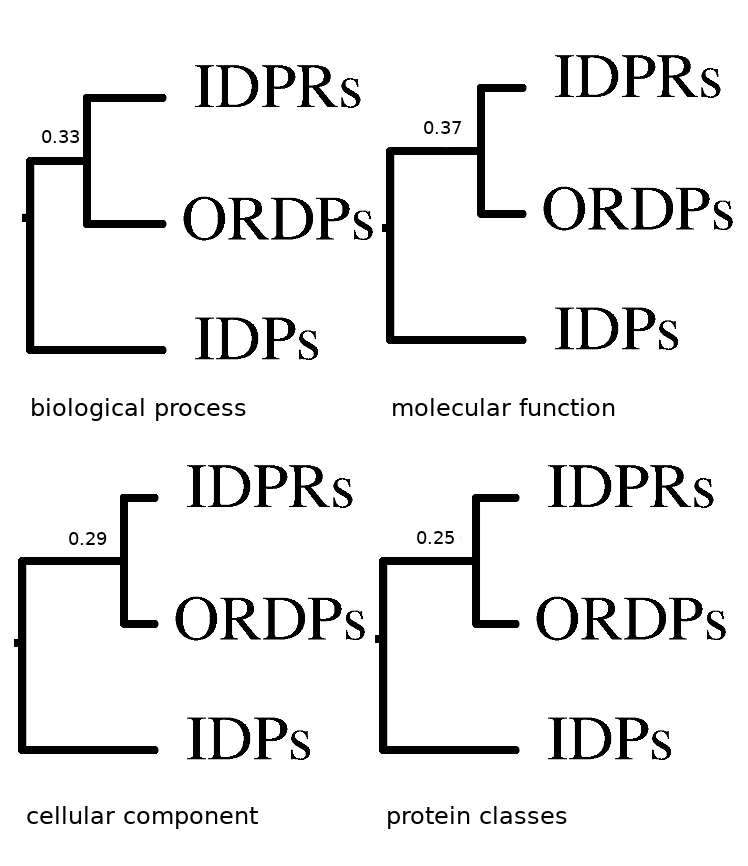


**Figure A: Hierarchical clusters of protein functional profiles for protein variants populated with proteins randomly selected from the human proteome.** To further check that ORDPs and IDPRs do not clustered together by chance, we compared the hierarchical clusters of ORDPs, IDPRs and IDPs with the clusters obtained by inserting in each variant (ORDPs, IDPRs and IDPs) proteins randomly selected from the human proteome and repeating this procedure 100 times. After this extra bootstrap we conclude that the lower distance between the functional profiles of ORDPs and IDPRs observed in the human proteome is significant. The numbers beside the branch of the trees connecting ORDPs and IDPRs represent the number of times these two groups clustered together in the 100-times replication bootstrap procedure.
